# Intravenous Everolimus Formulation (Sapu003) for Clinical Trials

**DOI:** 10.3390/ijms27135775

**Published:** 2026-06-26

**Authors:** Sheng-Hao Min, Kevin Forero, William Putnam, Jonathan Anderson, Robert Hoff, John Lopp, Vuong Trieu, Kwun Ho, Cynthia Lee

**Affiliations:** 1 Sapu Bioscience, LLC, 10840 Thornmint Road, Suite 118, San Diego, CA 92127, USA; jack.min@sapubio.com (S.-H.M.); kevin.forero@sapubio.com (K.F.); william.putnam@sapubio.com (W.P.); jonathan.anderson@sapubio.com (J.A.); robert.hoff@sapubio.com (R.H.); john.lopp@sapubio.com (J.L.); kwun.ho@sapubio.com (K.H.); 2 Oncotelic Therapeutics Inc., 29397 Agoura Road, Suite 107, Agoura Hills, CA 91301, USA; vtrieu@oncotelic.com; 3 Sapu Nano (US) LLC, 10801 Thornmint Rd. Suite 150, San Diego, CA 92127, USA

**Keywords:** Everolimus, Sapu003, nanoparticle, Deciparticle™, manufacturing

## Abstract

Everolimus is approved for the treatment of advanced renal cell carcinoma after VEGF-targeted therapy, metastatic HR-positive/HER2-negative breast cancer in combination with exemestane, and other oncologic indications. However, an intravenous option has not been developed, largely due to its pronounced hydrophobicity and limited oral bioavailability of approximately 15–20%. In this study, we report the development of Sapu003, a novel intravenous Everolimus formulation enabled through the Deciparticle™ platform. A diverse library of mPEG-based block copolymers was evaluated for their ability to encapsulate Everolimus and self-assemble into stable nanoparticle structures. mPEG-Chol was ultimately selected based on its favorable biocompatibility characteristics. In addition to Everolimus, mPEG-Chol and related analogs demonstrated broad formulation compatibility with multiple hydrophobic therapeutics, including Sirolimus, Tacrolimus, Cyclosporine, as well as representative peptides and polyketides. Clinical manufacturing was conducted in a cGMP environment over a 7-day production cycle. Production was carried out under amber light using light-protective vials to reduce drug degradation. The bulk material was sterile-filtered, and subsequent fill/finish/lyophilization operations were performed under temperature-controlled conditions with high precision in fill accuracy (≥98%). After reconstitution, the final product yielding uniform Deciparticles™ that met predefined sterility and particle size criteria. Stability studies demonstrated that the formulation remained stable for at least one month at 5 °C and retained acceptable in-use stability for at least 24 h at room temperature. The process was successfully scaled beyond 10 g, supporting an ongoing Phase 1b open-label dose escalation clinical study of Sapu003 in combination with exemestane in patients with advanced mTOR-sensitive solid tumors (NCT07369505). In vivo evaluation demonstrated strong antitumor efficacy following intravenous administration (QW × 3), with tumor growth inhibition reaching 97–98% in the U-87MG glioblastoma xenograft model. No evidence of phlebitis was observed with repeated tail vein dosing. In this model, Sapu003 dosed weekly showed superior tumor suppression compared with oral Everolimus. Collectively, screening of a mPEG-block copolymer library identified mPEG-Chol as a lead excipient capable of consistently forming stable Deciparticles™ with sub-20 nm mean particle size. The resulting intravenous Everolimus formulation demonstrated scalable manufacturing, favorable stability, and potent antitumor activity in preclinical models, supporting further clinical evaluation of Sapu003 in advanced solid tumors.

## 1. Introduction

Everolimus (marketed as Afinitor^®^ in oncology and Zortress in transplantation) is an orally bioavailable inhibitor of the mammalian target of Rapamycin (mTOR) with broad clinical applications in transplantation and oncology, and the Rapamycin class has also been widely applied in interventional cardiology through drug-eluting stent technologies. As a derivative of Sirolimus, Everolimus functions as a proliferation signal inhibitor within the mTOR inhibitor class and has been extensively reviewed in the contexts of renal transplantation, advanced renal cell carcinoma, and cardiovascular applications [1].

Everolimus is a highly hydrophobic macrocyclic mTOR inhibitor whose formulation for parenteral delivery is intrinsically challenging because of low aqueous solubility and limited solution stability. More broadly, rapalogs and other macrocyclic hydrophobic compounds frequently require carrier systems that simultaneously improve apparent solubility, maintain colloidal stability after dilution, and reduce dependence on aggressive surfactants or cosolvents [2]. Amphiphilic PEG-based di-block systems represent one of the most extensively studied strategies to address these limitations because they spontaneously assemble into core–shell nanostructures in aqueous media. In these systems, the hydrophobic domain acts as a reservoir for poorly soluble drug molecules while the hydrophilic PEG corona stabilizes the nanostructure and reduces nonspecific protein interactions in circulation [3].

Rao et al. described solvent-cast PEG-b-PLA micelles containing Everolimus and reported that the intrinsic aqueous solubility of Everolimus, approximately 9.6 μg/mL, increased to about 1.85 mg/mL after micellar incorporation [4]. These micelles exhibited mean hydrodynamic diameters below 50 nm, retained at least 97% of encapsulated Everolimus within solution over 48 h at room temperature, and demonstrated first-order diffusion-controlled release behavior [4]. These findings demonstrate that PEG di-block micelles can convert Everolimus from a practically insoluble compound into a nanoscale injectable formulation with therapeutically relevant drug concentrations.

A related study by Mishra et al. further demonstrated that Everolimus-loaded PEG-b-PLA micelles could be prepared at concentrations of approximately 2.0 mg/mL with particle diameters below 30 nm [5]. Drug release remained diffusion-controlled, and the micellar formulation preserved anti-proliferative activity against human umbilical vein endothelial cells. In vivo studies also showed that the micellar formulation had a maximum tolerated dose of approximately 50 mg/kg in mice [5]. Together, these studies provide direct evidence that PEG-based polymeric micelles represent a viable nanocarrier platform for Everolimus delivery.

Everolimus has also been incorporated into PEG–lipid micelles based on DSPE-PEG2000. Gianessi et al. reported that RAD001-loaded DSPE-PEG2000 micelles stabilized Everolimus under conditions in which the free drug was otherwise highly unstable in solution [6]. The formulation preserved more than 95% of activity after 14 days at 37 °C and remained stable during storage at 4 °C and 25 °C over the study period [6]. Although developed for intracerebroventricular administration rather than systemic oncology delivery, this work demonstrates that PEG–lipid micelles can significantly improve the solution stability of Everolimus and further supports PEG-based amphiphilic carriers as a rational formulation strategy for this class of macrocyclic mTOR inhibitors.

In the present work, we describe the development and application of a methoxy poly (ethylene glycol)–cholesterol (mPEG–Chol) amphiphilic carrier system for the intravenous formulation of Everolimus. This study focuses on the use of mPEG–Chol as a lipid–polymer hybrid excipient to generate stable nanoscale formulations of Everolimus suitable for parenteral administration. The hydrophobic cholesterol anchor promotes strong association within the micellar core, while the hydrophilic PEG corona provides steric stabilization in aqueous media and improves colloidal stability under physiologic dilution conditions. The resulting formulation, designated Sapu003 (Everolimus for Injection), represents a nanoscale micellar drug product currently in a Phase 1 clinical trial.

## 2. Results

### 2.1. Formulation Screening: Identification of mPEG-CONH-C18 and mPEG–Cholesterol (mPEG-Chol) as Candidate

#### 2.1.1. mPEG-CONH-C18 Versus mPEG–Chol

**Figure 1:**
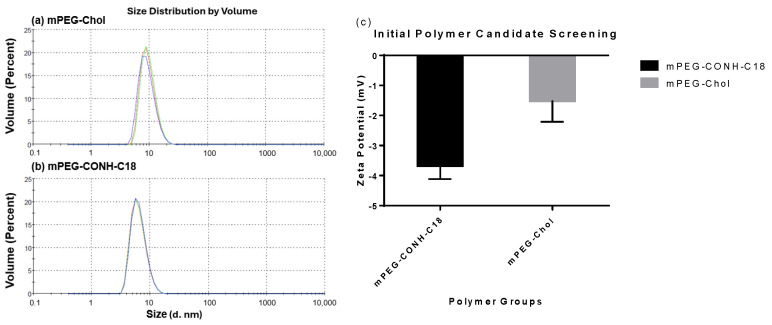
Mean particle size and zeta potential measurements during polymer screening (**a**) Mean particle size measurements for mPEG-Chol group; (**b**) Mean particle size measurements for mPEG-CONH-C18 group; (**c**) Zeta potential measurements for mPEG-Chol versus mPEG-CONH-C18.

Both maintained particle sizes around 12 nm within the target range for at least 24 h at room temperature (Figure 1).

mPEG-block polymers mPEG-CONH-C18 and mPEG–Chol were compared for their ability to form and stable Everolimus-loaded nanoparticles. Dynamic light scattering analysis demonstrated that both mPEG-CONH-C18 and mPEG–Chol formulations exhibited mean particle diameters of approximately 12 nm in mean particle diameters and remained stable for at least 24 h. Zeta potential measurements were also taken at the initial time points, showing values of around −3.67 mV for the mPEG-CONH-C18 formulation and −1.518 mV for the mPEG–Chol formulation.

**Figure 2:**
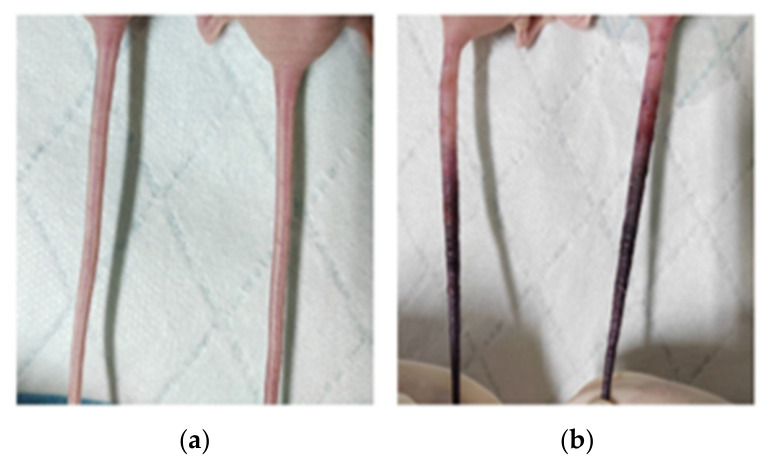
Preliminary animal safety evaluation. (**a**) Mice treated with the mPEG-Chol formulation demonstrated good local tolerability. In contrast, as shown in (**b**) Mice treated with the mPEG-CONH-C18 formulation exhibited acute injection-site toxicity.

During the initial in vivo safety assessment, tumor-bearing mice were randomized into groups to receive either C18-based or Chol-based formulations. As shown in Figure 2, the C18-based injection group exhibited significant local toxicity, characterized by severe tail necrosis at the injection site. In contrast, the Chol-based formulation was safe with no evidence of injection site toxicities.

Tumor-bearing mice were randomly assigned to receive either the mPEG-CONH-C18-based formulation or the mPEG-Chol-based formulation. As shown in Figure 2a, mice treated with the mPEG-Chol-based formulation demonstrated good local tolerability. In contrast, as shown in Figure 2b, mice treated with the C18-based formulation exhibited acute injection site toxicity.

#### 2.1.2. Defining the Formulation Space

**Figure 3:**
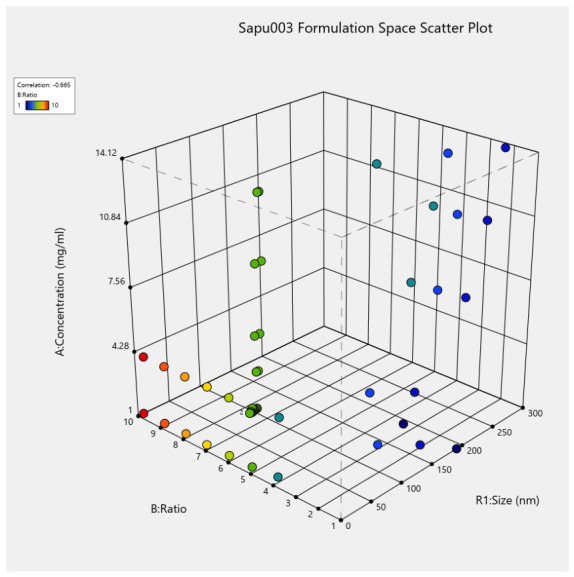
Formulation space identification byDoE. Particle size (R1, nm) was plotted on the *X*-axis as a function of concentration (A, mg/mL; vertical axis) and ratio (B; horizontal axis), with point color encoding ratio values (scale: 1–10). Each point represents an individual experimental run. A strong inverse relationship between ratio and particle size is observed (correlation = −0.665), with higher ratios (red/orange region) consistently associated with smaller particle sizes, while lower ratios (blue region) produce larger particles. At low ratios (B ≤ 3), particle sizes frequently exceed 150–300 nm across the full concentration range, indicating an aggregation-dominated regime. In contrast, at higher ratios (B ≥ 5), particle sizes collapse into the nanoscale domain (<50 nm), demonstrating a transition to a stabilized formulation regime. Concentration exerts a secondary effect, with increasing concentration contributing to moderate size expansion within the stabilized region but not inducing large-particle formation at sufficiently high ratios.

The study evaluated the effect of four formulation variables on particle size: concentration (1.00–14.12 mg/mL), drug-to-polymer ratio (1.00–10.00), ethanol (0–6.25%), and lactose (0–5.00%) (Figure 3). The dataset contained 49 observations. Across the 49 observations, particle size ranged from 7.8 to 288.47 nm, with a mean of 71.25 nm and a standard deviation of 91.29 nm, indicating a very broad and heterogeneous formulation space. The ANOVA (Table 1) identified ratio as the only statistically significant main effect at the conventional 0.05 threshold. Ratio had an F-value of 6.42 and *p* = 0.0155, whereas concentration showed a borderline effect (*p* = 0.0575), and the concentration × lactose interaction was also near significance (*p* = 0.0620). Ethanol, lactose, and the remaining two-factor interactions did not reach statistical significance individually in the 2FI model. Increasing ratio generally reduced particle size. From a formulation development perspective, the data suggests the existence of at least two distinct response regimes. One regime produced compact nanosized particles, generally in the ~8–20 nm range and was associated with higher ratios and lower-to-moderate drug concentrations. The other regime produced grossly enlarged particles, typically >90 nm and often >200 nm, and was associated with low ratios, especially when concentration increased. Intermediate formulations, particularly around ratio 5 with modest ethanol and lactose, gave particle sizes in the ~18–30 nm range, suggesting a transition zone between these two regimes. At drug-to-polymer ratio of 5 represents a critical threshold that defines entry into a robust nanoscale formulation space. Within this regime, concentration becomes the primary factor influencing particle size, producing a controlled and gradual increase in size with increasing drug load, while ethanol plays a secondary role and lactose contributes to enhanced stabilization.

Formulations at 1:5 through 1:10 maintained consistent particle sizes in the range of ~12–13 nm over the 24 h period, indicating improved stability and robustness compared to the 1:4 condition. The estimated mass fraction of API in final dosage form was calculated using the formula: API Mass % = Encapsulated API (mg)/Total formulation weight (mg) × 100% at the desired ratio of 1:5, the API mass percentage was around 16.7%. Drug loading was deemed as 100% as no Everolimus was precipitated out the solution. Encapsulation efficiency was not examined in this experiment, as the Everolimus intrinsic solubility in water is negligible, and any unbounded Everolimus would precipitate out from the solution and reflect on the increase in mean particle sizes.

### 2.2. Chemical Space: Defining the Deciparticle™ Chemical Space

**Figure 4:**
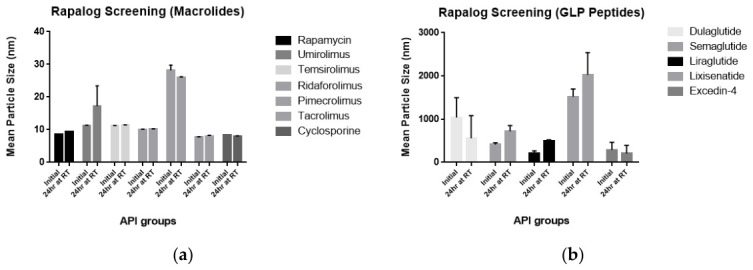
mPEG-Chol Deciparticle™ chemical space exploration. mPEG-Chol polymers were evaluated against different compounds using the screening method mentioned in Section 2.1. (**a**) Formulation screening with Rapamycin, Umirolimus, Temsirolimus, Ridaforolimus, Tacrolimus and Cyclosporine were able to be formulated by mPEG-Chol with mean particle sizes less than 20 nm. Pimecrolimus was also formulable, but sizes were larger than 20 nm. (**b**) Linear peptides and fatty acid modified peptides like Dulaglutide, Semaglutide, Liraglutide, Lixisenatide and Excedin-4 were not able to form nanoparticles smaller than 200 nm with mPEG-Chol.

**Figure 5:**
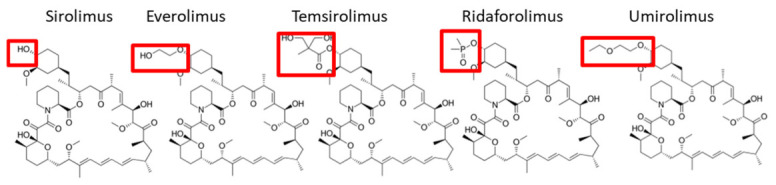
Rapamycin macrolide modifications. Sirolimus (C-40/42-OH), Everolimus (42-O-(2-hydroxyethyl), Temsirolimus (C-40/42 ester with 2,2-bis(hydroxymethyl)propionate), Ridaforolimus (C-40/42 O-phosphinate (dimethylphosphinate) ester), and Umirolimus (42-O-(2-ethoxyethyl) all formed stable Deciparticle™. The substituents do not have an impact Deciparticle™.The red box indicate the structure differences between each molecules.

To define the chemical space compatible with the mPEG-Chol Deciparticle™ nanocarrier platform, a series of structurally related peptidic and macrocyclic therapeutics were evaluated for their ability to form stable nanoparticle formulations (Figure 4). The test set was selected to cover several classes of compounds with increasing structural diversity, including Rapamycin-derived macrolide lactones (rapalogs), Ascomycin-derived macrolactams, cyclic peptides, linear peptides, and chemically modified peptides. Members of the Rapamycin macrolide family (rapalogs) consistently formed stable Deciparticle™ formulations. These molecules share a large hydrophobic macrocyclic lactone core (~31-membered ring) with variable substitutions at the C-40/C-42 positions. The following rapalogs were evaluated: Sirolimus (Rapamycin)—Native C-40/C-42 hydroxyl groups; Everolimus-42-O-(2-hydroxyethyl) substitution; Temsirolimus-C-40/C-42 ester with 2,2-bis(hydroxymethyl)propionate; Ridaforolimus-C-40/C-42 O-phosphinate (dimethylphosphinate) ester; and Umirolimus (Biolimus)-42-O-(2-ethoxyethyl) substitution (Figure 5).

Despite significant variation in side chain polarity and steric characteristics, all compounds successfully formed stable nanoparticles with the mPEG-Chol. These observations indicate that substituent variation at the C-40/C-42 position does not significantly impact nanoparticle formation, suggesting that the hydrophobic macrocyclic scaffold is the dominant structural.

**Figure 6:**
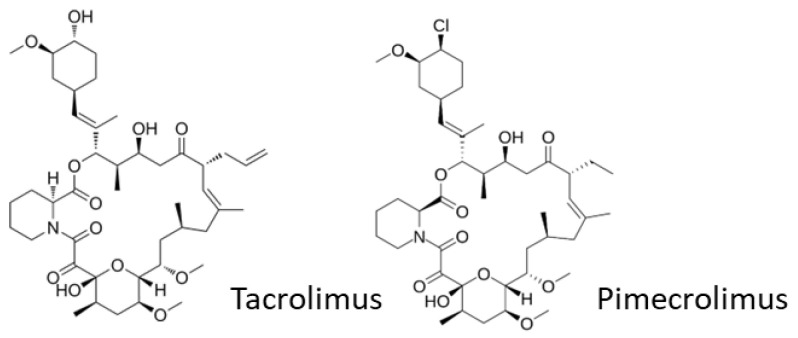
Ascomycin macrolactam. Tacrolimus (33-OH) and Pimecrolimus (33-epi-chloro-33-desoxyascomycin) are formulable but Pimecrolimus size exceeds that of Deciparticle™ (>20 nm).

To determine whether the Deciparticle™ system could accommodate macrocyclic structures beyond rapalogs, compounds from the Ascomycin macrolactam family were also evaluated (Figure 6). Tacrolimus (FK506-33-hydroxyl macrolactam) and Pimecrolimus (33-epi-chloro-33-desoxyascomycin). Tacrolimus formed stable nanoparticles with the mPEG-Chol, demonstrating that macrocyclic lactams with physicochemical properties similar to rapalogs also lie within the compatible chemical space. Pimecrolimus associated with the nanocarrier but produced particles larger than the optimal Deciparticle™ size range, indicating that increasing molecular bulk may approach the upper boundary of the compatible structural space.

**Figure 7:**
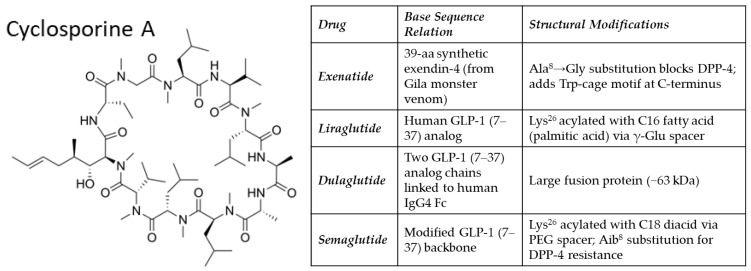
Peptides. Cyclosporine A (cyclic peptide) and Exenatide (synthetic linear peptide) are formulable. However fatty acid modified peptides or fusion peptides are not formulable.

To further define the structural boundaries of the Deciparticle™ mPEG-Chol formulation platform, the cyclic peptide (Figure 7): Cyclosporine was evaluated as a representative of hydrophobic cyclic peptides. Unlike the previously examined Rapamycin macrolides and Ascomycin macrolactams, Cyclosporine is not a macrolide but rather a cyclic undecapeptide characterized by extensive N-methylation of backbone amide nitrogens. This structural feature results in a peptide architecture that differs substantially from conventional linear peptide therapeutics. The N-methylation pattern reduces hydrogen bond donor capacity and increases the overall hydrophobic character of the molecule. Consequently, Cyclosporine presents a compact, conformationally restricted macrocycle with a predominantly hydrophobic molecular surface.

To evaluate whether peptide therapeutics could be incorporated into the mPEG-Chol Deciparticle™ nanocarrier system, a series of clinically relevant GLP-1–based peptide drugs were tested for their ability to form stable nanoparticles. The compounds selected represent the major classes of GLP-1 receptor agonists currently used clinically and differ primarily in the structural modifications introduced to extend plasma half-life.

The evaluated compounds included Exenatide, Liraglutide, Dulaglutide, Semaglutide (Figure 7). These molecules share related peptide backbones but contain distinct structural modifications such as fatty acid acylation, PEG linkers, Fc-fusion domains, or hybrid peptide sequences.

Exenatide, a 39 amino acid synthetic form of Exendin-4 derived from Gila monster venom, was successfully incorporated into the Deciparticle™ nanocarrier. The peptide contains relatively minimal structural modifications compared with next-generation GLP-1 agonists. Its primary modification is an Ala8 → Gly substitution that confers resistance to DPP-4 degradation, along with a Trp-cage motif at the C-terminus that stabilizes peptide folding. Under the mPEG-Chol formulation conditions, Exenatide formed stable Deciparticle™ nanoparticles within the expected particle size range, demonstrating that certain compact peptide scaffolds remain compatible with the platform.

In contrast to Exenatide, several GLP-1 receptor agonists containing structural modifications designed to prolong systemic half-life were not compatible with the Deciparticle™ system. Liraglutide is a human GLP-1 (7–37) analog modified through Lys26 acylation with a C16 fatty acid (palmitic acid) via a γ-Glu spacer. Despite being only modestly larger than Exenatide, Liraglutide did not produce stable nanoparticles. The lipid conjugation that promotes albumin binding in vivo likely alters the hydrophobic interaction pattern required for stable nanoparticle assembly. Dulaglutide consists of two GLP-1 analog chains fused to a human IgG4 Fc domain, producing a large fusion protein (~63 kDa). The molecular size of Dulaglutide greatly exceeds the size range of molecules successfully incorporated into the Deciparticle™ architecture. As a result, no stable nanoparticle formation was observed, indicating that large protein fusion constructs lie outside the compatible chemical space of the platform. Semaglutide is a modified GLP-1 analog containing Lys26 acylation with a C18 fatty diacid, a PEG spacer linker, Aib8 substitution for DPP-4 resistance. This molecule did not produce stable Deciparticle™ nanoparticles. The combination of PEG spacer insertion and lipid conjugation likely increases conformational flexibility and hydrophilicity, reducing the ability of the peptide to interact with the hydrophobic domains of the mPEG-Chol polymer.

The logP was used to determine whether hydrophobicity was the main cause for formulation compatibility. The data are shown in Table 2. In general, logP was consistent with ability to formulate; however, Exenatide has logP consistent of water-soluble molecules and yet was able to be formulated. Therefore, for peptide, the rules may be different.

### 2.3. Plasma Protein Binding of Sapu003

After 5 h incubation at 37 °C, Everolimus remained associated with the plasma with no detectable unbound Everolimus. Across CD-1 mouse, SD rat, beagle dog, cynomolgus monkey, and human plasma, protein binding exceeded 99.6% at 0.200 μM and exceeded 99.9% at both 2.00 and 20.0 μM. No meaningful species differences were observed. At all concentrations, the measured free Everolimus concentration in the ultrafiltrate remained below the assay quantitation limit (<1.00 nM) or near the lower boundary of quantitation. Consequently, the free Everolimus concentration was less than 0.000958 μg/mL (<0.000001 mg/mL), indicating that greater than 99.9% of the administered drug remained associated with plasma constituents. This behavior was maintained across a 100-fold concentration range spanning 0.200–20.0 μM. Across all concentrations, the unbound fraction remained below the analytical quantitation threshold, showing that the solubility limit of Everolimus is extremely low—less than (<0.000001 mg/mL).

### 2.4. Progression from R&D to Clinical Manufacturing

**Figure 8:**
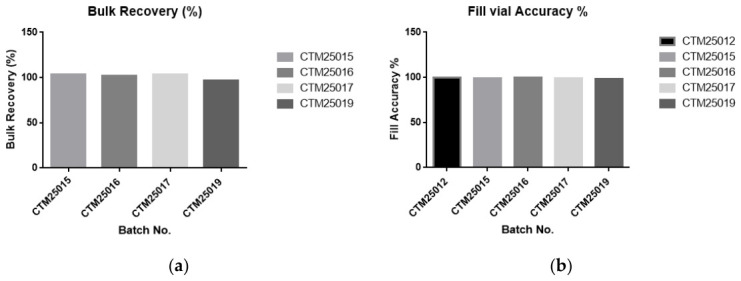
Progression from R&D to manufacturing. (**a**) Bulk recovery and assay percentages were determined by HPLC to validate production performance. The bulk recovery rate was consistently greater than 96% for all tested batches (CTM25012 was not evaluated for bulk recovery). Release testing for assay percentage showed that CTM25012 had an assay of 88%, which was attributed to filling based on the theoretical concentration without prior bulk concentration verification. (**b**) Filling accuracy: Beginning with CTM25015, bulk concentration was tested prior to filling, and the resulting assay values were consistently greater than 94%.

To advance toward clinical-ready manufacturing, three clinical batches of Sapu003 were produced at increasing scales within our ISO 5/GMP [7,8]-certified facility in San Diego, CA, USA. Following process optimization, three larger-scale batches were produced, each at a scale of 12 g Everolimus. Bulk concentrations were analyzed by HPLC to validate production quality across batches, with recoveries consistently greater than 96% (Figure 8a). Filling accuracy was also assessed by weight during the fill-and-finish process, demonstrating consistently greater than 98% accuracy.

### 2.5. Characterization: Release Testing and Colloidal Stability

Release and storage stability testing were performed on three cGMP-manufactured batches, with summarized results presented in Table 3. From release tests, all batches demonstrated comparable product characteristics, including Everolimus assay values ranging from 94.5% to 98.4%, pH values ranging from 5.0 to 5.2, and water content between 1.4% and 1.6%. Reconstituted particle sizes were consistent across batches, ranging from 13.1 to 13.9 nm, while residual ethanol levels ranged from 75.6 to 115.7 ppm. Collectively, these findings indicate a robust and reproducible manufacturing process with consistent product quality across batches. Long-term storage stability testing demonstrated that all three batches maintained acceptable Everolimus purity when stored at 2–8 °C for at least six months. In contrast, elevated temperature and humidity conditions resulted in accelerated degradation of Everolimus. After six months of storage at 25 °C/60% RH ± 5% RH, assay values decreased by approximately 5%, whereas storage at 30 °C/65% RH ± 5% RH resulted in assay losses exceeding 30% across all batches. These findings highlight the importance of refrigerated storage for maintaining product stability.

Residual ethanol content in Sapu003 (Everolimus for Injection) was determined as part of release testing using a validated static headspace gas chromatography method with flame ionization detection (HS-GC/FID). Analyses were performed on an Agilent 8890 gas chromatograph (Agilent Technologies, Santa Clara, CA, USA) equipped with a 7697A headspace sampler (Agilent Technologies, Santa Clara, CA, USA)and an HP-5 capillary column (30 m × 0.32 mm, 0.25 μm film thickness) (Agilent Technologies, Santa Clara, CA, USA). Lyophilized drug product samples were reconstituted to 5 mL and prepared in dimethyl sulfoxide (DMSO) prior to analysis. Ethanol calibration standards were prepared at nominal concentrations of 3.1, 5.1, and 7.1 mg/mL and analyzed alongside test samples. Quantitation was performed using an external standard calibration curve with the regression equation y = 1.3314x and a correlation coefficient (R^2^) of 0.99998. System suitability was demonstrated through six replicate standard injections, yielding a peak-area relative standard deviation (RSD) of 0.432% and retention time RSD of 0.081%, both meeting the predefined acceptance criterion of ≤2.0%.

Ten representative Sapu003 drug product vials (QCIDs H250715–H250724) were analyzed in triplicate. Residual ethanol concentrations were calculated from chromatographic peak areas and expressed as parts per million (ppm). Mean residual ethanol levels were less than 200 ppm across individual vials. Replicate analyses demonstrated excellent precision, with peak-area RSD values ranging from 0.059% to 0.730%. All measured ethanol levels were substantially below the USP <467> and ICH Q3C Class 3 solvent acceptance limit of 5000 ppm. These results demonstrate efficient removal of ethanol during the manufacturing and lyophilization processes.

**Figure 9:**
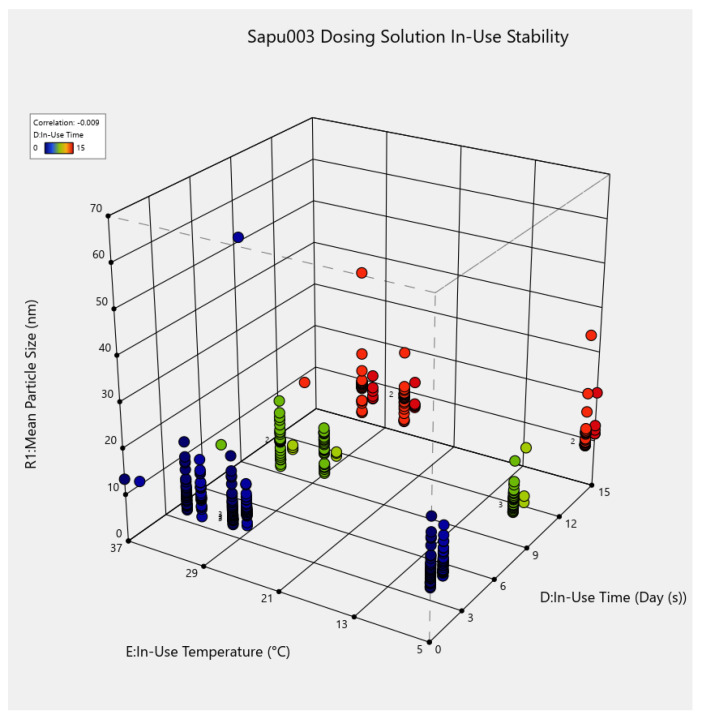
Effect of in-use time and temperature on mean particle size of Sapu003 dosing solution. Three-dimensional scatter plot shows mean particle size (nm) as a function of in-use time (days, D) and in-use temperature (°C, E). Each point represents an individual experimental observation across multiple lots and storage conditions. Data points are color-coded by in-use time (0–15 days), as indicated by the color scale. Overall correlation between in-use time and particle size is minimal (r = −0.009), indicating no simple monotonic relationship. While many observations remain within the nanoscale range ~10–20 nm).

Sapu003 in-use stability study was built as a response surface, split-plot design using imported data with 444 runs and a reduced quadratic model plus user-added terms (Figure 9). The factors were lot identity as a four-level categorical variable: lot number, storage time from 0 to 6 months, storage temperature from 5 to 30 °C, in-use time from 0 to 15 days, and in-use temperature from 5 to 37 °C. Across those modeled observations (417 observations), mean particle size ranged from 5 to 390.9 nm, with an overall mean of 14.88 nm and standard deviation of 19.06 nm. A central strength of this dataset is that it examines both long-term storage stress and post-reconstitution or in-use stress in a single multivariable framework. The most important result is that in-use conditions were more influential than storage duration. In-use time was the dominant main effect, with F = 41.04 and *p* < 0.0001, followed by in-use temperature with F = 13.48 and *p* = 0.0003. Storage temperature was also significant, though less strongly, with F = 4.91 and *p* = 0.0272. By contrast, storage time as an isolated linear term was not significant, with *p* = 0.2963. The main-effect coefficients for in-use time, in-use temperature, and storage temperature are all negative, indicating that increasing these factors is associated with a reduction in particle size under the linear model (Table 4). This pattern indicates that particle size in Sapu003 was highly stable with trend to reduction in size in time and temperature in use.

### 2.6. In Vivo Biological Activities: Safety and Efficacy Evaluation

**Figure 10:**
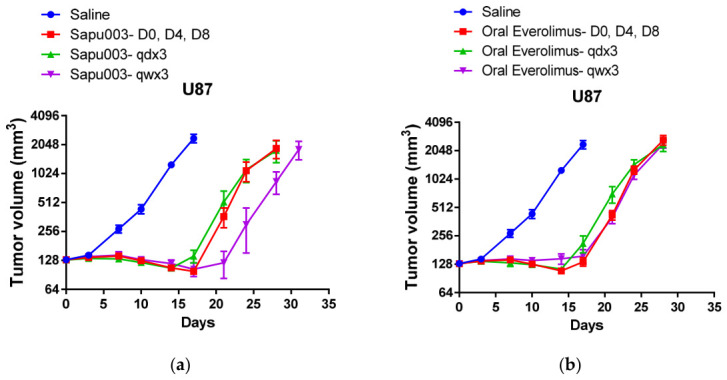
The effects of Sapu003 on tumor volume in U-87 MG xenograft model. (**a**) Tumor volume monitoring for Sapu003 treatment groups. (**b**) Tumor volume monitoring for oral Everolimus treatment groups. Tumor-inoculated mice were randomized into treatment groups and received their respective interventions beginning on Day 0. Sapu003 was administered intravenously at a dose of 30 mg/kg for three different dosing schedules: three consecutive days (QD × 3), every four days D0, D4, D8 × 1 and weekly QW × 3. Oral Everolimus was administered at the same dosing schedule as the comparison group. Saline was administered intravenously once daily for three weeks (QW × 3) and served as the control group. Tumor sizes were measured twice a week. The saline group showed an increase in tumor volume with the increase in time, Sapu003 treatment showed the significant inhibition of tumor growth compared with the control group (*p* < 0.001, two-way ANOVA). Each value was presented as mean ± SEM from 7 mice per group. At Day 28, treatment with Sapu003 (30 mg/kg, IV, QW × 3) resulted in statistically significant superiority over all oral Everolimus treatment groups (*p* < 0.05).

Tumor-inoculated mice were randomized into treatment groups and received their respective interventions beginning on Day 0. Tumor-inoculated mice were randomized into seven groups and received different treatment on Day 0. PEG-400 Everolimus (Oral Everolimus) was administered orally (PO) at a dose of 30 mg/kg, with dosing frequencies of D0, D4, D8 × 1, QD × 3 and QW × 3. Sap003 was administered intravenously (IV) at a dose of 30 mg/kg, with dosing frequencies of D0, D4, D8 × 1, QD × 3 and QW × 3. Saline was used as the control group, administered IV once weekly for three weeks (QW × 3). After Day 17, mice from the saline control group were removed from study and euthanized due to large tumors, so the data from Day 17 was selected for analysis in this section. Figure 10 summarized the results of the tumor volume and the change in tumor growth in the U-87 MG xenograft model. The average tumor volume for all groups on Day 0 was around 130 mm^3^, and the average tumor volume of the saline group was 2379.94 ± 240.18 mm^3^ on Day 17. The tumor volume of the oral Everolimus (D0, D4, D8 × 1, QD × 3 and QW × 3) and Sapu003 (D0, D4, D8 × 1, QD × 3 and QW × 3) groups were 135.74 ± 13.99 mm^3^, 212.15 ± 43.16 mm^3^, 155.72 ± 27.18 mm^3^, 98.47 ± 27.18 mm^3^, 141.96 ± 21.56 mm^3^ and 103.91 ± 16.81 mm^3^ on Day 17, which showed a significant tumor inhibition effect compared to the saline group (*p* < 0.001). In Sapu003 groups, superior efficacy was recorded for QW × 3 dosing versus QD × 3 dosing and D0, D4, D8 × 1 dosing. At Day 28, the tumor volume in the Sapu003 (30 mg/kg, IV, QW × 3) group was 844.78 ± 221.28 mm^3^, whereas the tumor volumes in the oral Everolimus (D0, D4, D8 × 1); QD × 3; and QW × 3) groups were 2648.44 ± 310.96 mm^3^, 2370.11 ± 367.40 mm^3^, and 2411.32 ± 228.28 mm^3^, respectively. Treatment with Sapu003 (30 mg/kg, IV, QW × 3) demonstrated statistically significant improvement over all oral Everolimus treatment groups (*p* < 0.05).

## 3. Discussion

### 3.1. Formulation Screening: Identification of mPEG-CONH-C18 and mPEG–Cholesterol (mPEG-Chol) as Candidates

#### 3.1.1. Polymer Candidate Identification and Failure of Fatty Acid C18-mPEG Polymer

Our investigation into polymer selection for the Sapu003 pipeline focused on identifying candidates capable of forming small, stable Everolimus-loaded Deciparticle™. Among the screened polymers, nine were able to successfully form stable Deciparticles™. Based on particle size, stability profile, and overall formulation performance, mPEG-Chol (mPEG–Cholesterol) and of mPEG-CONH-C18 were selected for further evaluation and development. However, the in vivo tolerability of the initial mPEG-CONH-C18-based formulation was evaluated in tumor-bearing mice, significant local toxicity was observed. Mice developed severe tail necrosis at the injection site, indicating substantial local vascular or perivascular damage associated with the formulation. Given the extent of tissue damage and the implications for clinical translatability, the mPEG-CONH-C18 formulation was deemed unsuitable for further development. As a result, polymers containing aliphatic long-chain fatty acid components were eliminated from subsequent screening efforts. On the other hand, mPEG-Chol(mPEG–Cholesterol)-based formulation demonstrated good injection site tolerability. Based on the in vivo tolerability findings, subsequent formulation development efforts were focused on the mPEG-Chol-based platform for Sapu003 Deciparticles™.

Amphiphilic polymers containing a hydrophilic polyethylene glycol (PEG) segment and a hydrophobic C18 lipid-derived moiety have been widely investigated as carriers for poorly water-soluble drugs. These systems typically self-assemble into micelles with a hydrophobic core capable of incorporating lipophilic payloads and a PEG shell that improves aqueous dispersibility and colloidal stability. Examples include PEG–C18 micelles for lipophilic gemcitabine prodrugs and PEG-block-poly (N-hexyl stearate L-aspartamide) micelles for amphotericin B [9,10]. Zhu et al., who developed acid-sensitive PEG–C18 micelles for delivery of a lipophilic gemcitabine prodrug (GemC18). In that study, the PEG–C18 micellar system significantly improved antitumor activity in B16-F10 melanoma-bearing mice, demonstrating the ability of PEG/C18 amphiphiles to enhance delivery and efficacy of highly hydrophobic antitumor payloads in vivo [9]. Lavasanifar et al. demonstrated that these PEG/C18 micelles efficiently incorporated amphotericin B, improved apparent solubility, and reduced hemolytic toxicity in vitro [11]. In a subsequent study, Diezi et al. evaluated the same micellar platform in rats and reported favorable pharmacokinetic behavior together with reduced nephrotoxicity compared with conventional amphotericin B formulations, supporting the in vivo advantages of this PEG–C18 micellar carrier [10]. PEG/C18-type systems have also been applied to other poorly soluble anticancer agents. Dong et al. reported mPEG-SS-2SA/TPGS mixed micelles for paclitaxel delivery, where a mPEG–stearate-type amphiphile formed reduction-responsive micelles that promoted intracellular drug release and reversed multidrug resistance in tumor cells in vitro [12]. Similarly, Guan et al. described stearic-acid-modified polysaccharide micelles for docetaxel delivery, which demonstrated efficient drug loading, cellular uptake, and in vitro anticancer activity [13]. These studies further support the utility of C18-bearing amphiphiles for solubilizing highly hydrophobic taxanes.

However, on our hand the mPEG-CONH-C18 proven to be locally necrotic and was not suitable for additional formulation development. This branch of di-block polymer was eliminated early on during the development of Everolimus for Injection. The cause of this tail necrosis has not been further investigated.

Methoxy poly (ethylene glycol)–cholesterol (mPEG–Chol) is an amphiphilic conjugate composed of a hydrophilic polyethylene glycol (PEG) chain covalently linked to a hydrophobic cholesterol moiety. This architecture enables spontaneous self-assembly in aqueous media into nanoscale core–shell micelles, with a hydrophobic cholesterol-rich core surrounded by a hydrated PEG corona. Such amphiphilic structures are particularly attractive for intravenous delivery of poorly water-soluble drugs because they provide a mechanism for solubilizing lipophilic compounds while maintaining colloidal stability in physiological environments [14]. PEGylation has been shown to improve colloidal stability, reduce uptake by the mononuclear phagocyte system and prolong systemic circulation time for nanocarriers in vivo [15]. Consequently, PEG–Chol conjugates have been investigated as components of several drug delivery systems including micelles, vesicles, and PEGylated lipid carriers.

An important physicochemical characteristic of PEG–Chol systems is their ability to form micelles with very low critical micelle concentration (CMC). Low CMC values are desirable for intravenous formulations because injected micelles undergo rapid dilution in the bloodstream. Systems with sufficiently low CMC can maintain micellar integrity despite this dilution. PEG–Chol conjugates have been reported to exhibit CMC values in the micromolar range, enabling stable micelle formation even under highly diluted conditions [15]. Experimental work by Búzová et al. demonstrated that PEG–cholesterol micelles could effectively solubilize the hydrophobic photosensitizer hypericin and that micelle formation occurred near concentrations of approximately 10^−6^ M, supporting the notion that PEG–Chol micelles can remain intact at low concentrations [16]. Additionally, cholesterol is a rigid sterol that can enhance hydrophobic interactions and stabilize nanoscale assemblies. Evidence from related cholesterol-containing polymeric carriers supports the concept that cholesterol motifs can significantly increase loading capacity and kinetic stability for hydrophobic drugs. For example, cholesterol-containing biodegradable block copolymers have been reported to produce nanoscale micelles with high paclitaxel loading capacity and improved stability [17]. Although these systems are structurally more complex than simple mPEG–Chol conjugates, they reinforce the importance of cholesterol in promoting hydrophobic drug association.

#### 3.1.2. Drug Loading and Composition

From Figure 3, these results demonstrate that sufficient polymer content is critical for effective encapsulation and stabilization of Everolimus within the micellar system. Lower ratios (≤1:3) failed to provide adequate polymer coverage, resulting in precipitation or formation of oversized particles. While a ratio of 1:4 was capable of initially forming appropriately sized particles, the observed increase in particle size over time suggests marginal stability, indicating that this ratio represents a borderline condition. Ratios of 1:5 and above provided consistently small and stable particle sizes, reflecting more complete encapsulation and improved micelle integrity. Based on these findings, a 1:5 API-to-polymer ratio was selected as the optimal condition, balancing formulation stability, particle size control and efficient polymer utilization for further development.

### 3.2. Unique Chemical Space for mPEG-Chol (mPEG–Cholesterol)

The present study experimentally defines the structural chemical space compatible with the mPEG-Chol Deciparticle™ nanocarrier platform. By evaluating a series of structurally related therapeutic compounds, clear boundaries emerged regarding which molecular architectures can be successfully incorporated into stable nanoparticles.

The most striking observation was the consistent compatibility of Rapamycin-derived macrolide lactones. Rapalogs such as Sirolimus, Everolimus, Temsirolimus, Ridaforolimus, and Umirolimus all formed stable Deciparticle™ formulations despite substantial differences in their C-40/C-42 substituent groups. These results indicate that the macrocyclic lactone core and overall hydrophobic surface area dominate the interaction with the mPEG-Chol amphiphilic polymer, while peripheral substituent variations exert minimal influence on nanoparticle formation.

This finding has important implications for the development of Deciparticle™ formulations of mTOR inhibitors, particularly Everolimus, which is the active pharmaceutical ingredient in the Sapu003 intravenous formulation currently under development for clinical evaluation. The demonstrated tolerance for multiple rapalog derivatives suggests that the platform may be broadly applicable across this drug class without requiring extensive reformulation.

The results also show that Ascomycin-derived macrolactams such as Tacrolimus fall within the compatible structural envelope, further reinforcing the importance of large hydrophobic macrocycles as optimal payloads for the platform. However, the observation that Pimecrolimus produced particles exceeding the optimal size range suggests the presence of an upper steric limit for compounds that can be efficiently accommodated within the nanoparticle architecture.

Exenatide, a 39 amino acid peptide derived from Exendin-4, represents the least structurally modified molecule among the tested GLP-1 receptor agonists. Sequence-based hydropathy analysis indicates that Exenatide is overall hydrophilic, with a negative grand average of hydropathy (GRAVY) value consistent with the predominance of polar and charged amino acid residues. Despite this overall hydrophilic character, the peptide contains several hydrophobic amino acid residues, including leucine, isoleucine, valine, phenylalanine, and tryptophan, which create localized hydrophobic patches along the peptide surface.

These hydrophobic residues likely act as anchoring points for interaction with the hydrophobic domains of the mPEG-Chol polymer, allowing the peptide to associate with the nanocarrier core while the surrounding polar residues remain solvent-exposed. This amphipathic character may facilitate stable encapsulation within Deciparticle™ nanoparticles despite the overall hydrophilic nature of the peptide.

In contrast, other GLP-1 receptor agonists evaluated in this study contain structural modifications designed to prolong plasma half-life, including fatty acid acylation (Liraglutide), PEG spacer linkers (Semaglutide), or Fc-fusion domains (Dulaglutide). These modifications substantially alter the physicochemical architecture of the peptide and were not compatible with stable Deciparticle™ formation under the tested conditions. Collectively, these observations suggest that compact peptides with localized hydrophobic residues capable of acting as polymer interaction anchors remain compatible with the mPEG-Chol platform, whereas extensive half-life extension modifications disrupt nanoparticle assembly.

Within this framework, Cyclosporine represents a particularly informative example. Although it is formally a peptide, its cyclic structure and extensive N-methylation produce a compact and lipophilic macrocyclic architecture. These features reduce conformational flexibility and limit hydrogen-bonding interactions with the aqueous environment, thereby promoting hydrophobic surface exposure. Such properties are consistent with those observed for the macrolide and macrolactam drugs that were successfully incorporated into Deciparticle™ nanoparticles.

### 3.3. Plasma Protein Binding for Sapu003

The formulation was diluted directly into plasma and maintained at 37 °C for 5 h, allowing sufficient time for potential micelle dissociation, drug redistribution, or release to occur. If significant release of Everolimus had occurred, freely diffusible drug would have been expected to appear in the ultrafiltrate compartment and be quantifiable by LC-MS/MS. Instead, free Everolimus remained below the assay quantitation limit across all species and concentrations tested. Following dilution into plasma at concentrations ranging from 0.200 to 20.0 μM, no increase in measurable free Everolimus was observed. Sapu003/Everolimus for Injection demonstrated extremely high plasma association in mouse, rat, dog, monkey, and human plasma, with plasma binding exceeding 99.6% at 0.200 μM and exceeding 99.9% at 2.00 and 20.0 μM.

The assay lower limit of quantitation was 1.00 nM, corresponding to 0.000958 μg/mL (0.000000958 mg/mL). Free Everolimus concentrations remained below this threshold across all concentrations tested. These findings indicate that Sapu003 maintains a highly associated state following dilution into plasma and does not generate a measurable freely circulating Everolimus fraction under the conditions tested. The results support the plasma stability of the Deciparticle™ formulation and provide evidence that rapid dissociation or burst release does not occur upon exposure to biological fluids. This observed stability is likely related to the poor aqueous solubility of Everolimus.

### 3.4. Progression from Formulation to CTM Manufacturing

The progression of Sapu003 from early formulation development to CTM manufacturing was achieved through a structured and scalable process strategy. Initial development batches were filled manually using repeat pipettors within an ISO-5 laminar airflow environment (IsoTech Design Inc., Saint-Laurent, Canada) consistent with clinical manufacturing conditions. As demand expanded to support nonclinical toxicology and clinical trial material (CTM) production, the filling process was systematically scaled from the Dara SX-50 benchtop peristaltic pump (Dara Pharmaceutical Equipment, Granollers, Spain) to the Makwell fully automatic filling machine (Makwell, Wuxi, China). Throughout this transition—from manual pipetting to intermediate SX-50 pump filling and ultimately to full clinical-scale Makwell operations—critical process parameters, including filling speed, sterility assurance, fill weight accuracy, bulk solution homogeneity, and product temperature control, were maintained or improved. Clinical-scale batches consistently met > 98% of fill weight accuracy specifications, and sterility was confirmed both through product testing and robust environmental monitoring demonstrating sustained ISO-5 conditions.

The platform supports an integrated workflow from early formulation development through nonclinical toxicology supply and Phase 1 clinical trial manufacturing. Bulk drug substance is produced via a one-pot process, followed by aseptic fill finish and lyophilization to yield the final drug product. Quality control and quality assurance operations are supported by an AI-enabled data management system (DMS) and quality management system (QMS), enabling streamlined documentation and compliance. The facility is designed to support a development capacity of up to four new IND programs per year, demonstrating scalability and operational readiness for continued pipeline expansion.

### 3.5. Characterization: Release Testing and In-Use Stability

The comprehensive GMP release testing conducted on the final drug product demonstrates a robust quality control strategy, ensuring the formulation meets all critical quality attributes for Phase 1 clinical use.

Sapu003 is supplied as a lyophilized drug product, and all release and stability testing was performed following reconstitution. Reconstituted particle sizes remained highly consistent across clinical batches (13.1–13.9 nm at release) and remained within specification during long-term storage studies, demonstrating that the lyophilization process did not adversely affect nanoparticle integrity or induce significant particle aggregation. Everolimus assay results further supported the robustness of the manufacturing process, with average assay values exceeding 96.3% across tested batches. Measured pH values remained within the target range of 4–6, consistent with formulation stability and compatibility with intravenous administration. In addition, low residual water content less than 3% and residual ethanol levels below 200 ppm confirmed efficient lyophilization and supported the overall quality and stability of the drug product. Sterility and endotoxin testing confirmed that the product is safe for clinical administration, free from microbial contamination and pyrogenic risks. Stability assessment included both colloidal stability parameters (particle size) and chemical stability of the active pharmaceutical ingredient. Everolimus assay values were monitored during release and long-term storage studies. Refrigerated storage (2–8 °C) maintained acceptable Everolimus assay values for at least six months, whereas accelerated storage conditions produced progressive API degradation, confirming the sensitivity of the assay method to detect loss of drug stability.

Notably, Sapu003 demonstrated favorable storage stability under refrigerated conditions (2–8 °C) for at least six months. Reconstitution stability testing has demonstrated at least 15 days in use at room temperature highlights the formulation’s practical usability in a clinical setting, allowing flexibility in handling and dosing schedules. Overall, these results collectively demonstrate that the drug product is well-characterized, stable, and safe for Phase 1 clinical evaluation, providing confidence in both the manufacturing process and the product’s quality profile.

### 3.6. In Vivo Biological Activities: Safety and Efficacy Evaluation

The xenograft study highlights the therapeutic potential of Sapu003 across multiple tumor models with varying mTOR signaling profiles and metabolic dependencies. Among the models tested, U-87 MG, which exhibits high basal mTORC1 (p-S6K1, p-4E-BP1) and mTORC2 (p-AKT Ser473, p-SGK1) activity, showed pronounced sensitivity to Sapu003 and exceeded cytostatic effect into actual tumor-killing cytotoxicity, which may correlate with its high glycolytic and amino acid addiction. The reformulation of Afinitor^®^ (oral Everolimus) into Sapu003 (intravenous Everolimus) allows the full delivery of Everolimus to tissues without the 90% loss in the GI tract reducing the GI toxicity associated with Afinitor^®^. It also increases the tissue and tumor accumulation of Everolimus allowing for the observed tumor kill by Sapu003 that is effective with less frequent dosing. As shown in Figure 10, the antitumor activity was made more effective with the weekly dosing. The same was not observed for oral Everolimus. This aligns with the concept that tumors highly dependent on glycolysis and nutrient uptake are particularly vulnerable to mTORC1-targeted interventions combined with the Deciparticle™ delivery platform.

Sapu003 has advanced into an ongoing Phase 1b clinical trial following completion of pharmacology, pharmacokinetic, biodistribution, metabolism, excretion, tolerability, and toxicology studies. A key finding from the nonclinical program was that intravenous administration of Sapu003 substantially altered the tissue distribution profile relative to oral Everolimus. Unlike oral Everolimus, which demonstrated preferential accumulation within the gastrointestinal tract, Sapu003 exhibited rapid and broad systemic tissue distribution with markedly reduced deposition in the stomach and small intestine. Tissue distribution studies demonstrated that oral Everolimus accumulated predominantly in gastrointestinal tissues, whereas Sapu003 distributed more uniformly to highly perfused organs and peripheral tissues. Importantly, no intestinal toxicity was observed in the dog studies following intravenous administration of Sapu003. These findings support the underlying design rationale of Sapu003, namely, to bypass intestinal absorption, reduce gastrointestinal exposure, and avoid the high local gastrointestinal drug concentrations associated with oral administration [18].

Pharmacokinetic studies further demonstrated that intravenous Sapu003 achieved substantially greater systemic exposure than oral Everolimus while maintaining the native metabolic disposition of the molecule. In rats, Sapu003 produced approximately 55-fold higher Cmax and 6.5-fold higher AUC than oral Everolimus administered at the same dose. Similar improvements in systemic exposure were observed in dogs. Plasma, microsomal, and hepatocyte stability remained comparable to native Everolimus across multiple species, and elimination remained primarily biliary and fecal, consistent with the known pharmacology of Everolimus.

The starting clinical dose was selected based on the totality of the available pharmacokinetic, biodistribution, tolerability, and toxicology data, together with the extensive clinical experience accumulated with oral Everolimus. The ongoing SP-03-B101 Phase 1b study employs a conservative Bayesian Optimal Interval (BOIN) dose escalation design beginning at 5 mg/m^2^ administered as a 30 min intravenous infusion, with escalation to 7.5 and 10 mg/m^2^ only after review of emerging safety data. These dose levels are supported by the nonclinical exposure and safety data and remain within exposure ranges previously characterized for Everolimus.

## 4. Materials and Methods

### 4.1. Synthesis of mPEG–Cholesterol (mPEG-Chol)

Manufacturing of mPEG-Chol polymer GMP-grade mPEG-Chol was manufactured at kilogram scales. Methoxy poly (ethylene glycol)–cholesterol (mPEG–Chol) conjugates were synthesized by covalent coupling of monomethoxy poly (ethylene glycol) (mPEG) to cholesterol using established PEG–lipid conjugation strategies. PEG chains with molecular weights ranging from approximately 400–10,000 Da have been reported for PEG–Chol systems, and several linkage chemistries can be used to generate the amphiphilic conjugate depending on the desired stability and physicochemical properties of the carrier [15]. mPEG–Chol was synthesized using a carbodiimide-mediated esterification reaction. Upon completion of the reaction, the precipitated dicyclohexylurea (DCU) by-product was removed by filtration. The crude product was concentrated and purified by precipitation in cold diethyl ether, followed by repeated washing to remove unreacted cholesterol and residual reagents. The purified mPEG–Chol conjugate was dried under vacuum to obtain a white amphiphilic polymer, consistent with previously reported PEG–cholesterol conjugates [19,20].

### 4.2. Chemicals Sources

mPEG-C18 polymer was obtained from Creative Pegworks, Durham, NC, USA. Everolimus, Temsirolimus, Ridaforolimus, Umirolimus, Pimecrolimus, Exendin-4, Dulaglutide, Semaglutide, Liraglutide, and Lixisenatide were purchased from MedChemExpress, Monmouth Junction, NJ, USA. Rapamycin, Tacrolimus, Cyclosporine A were purchased from Thermo Fisher Scientific, Waltham, MA, USA.

### 4.3. Formulation

The formulation was created using the thin film method. API compounds were dissolved by organic solvents (acetone or absolute ethanol (200-proof) (Thermo Fisher Scientific, Waltham, MA, USA) at the concentration of 2 mg/mL. Polymer was dissolved by matching organic solvent at 20 mg/mL each. For the polymer–API mixture, the polymer solution was combined with the API solution in a 1:1 (*v*/*v*) ratio, followed by intensive vortex for 10 s and incubation on a 60 °C heat block for 10 min or until organic solvent fully evaporated. Evaporation of acetone is facilitated using a vacuum pump to produce a film of API–polymer, with solvent removal aided by blowing air generated by the pump for roughly 3–5 min per sample, ensuring the replacement of the pump tip for each solution. Finally, samples were incubated at room temperature for 15 min and reconstituted with deionized water. The hydrodynamics diameter was performed using the Malvern Zetasizer 3600 (ZS-3600) (Malvern Panalytical, Westborough, MA, USA) and Wyatt Dynapro Plate Reader II (Waters | Wyatt Technology Corporation, Goleta, CA, USA).

Particle size and polydispersity index (PDI) of Sapu003 formulations were determined using dynamic light scattering (DLS). Measurements were performed using a calibrated DLS instrument: Malvern Zetasizer ZEN3600 and Wyatt Dynapro Plate Reader II under controlled temperature conditions. The samples were diluted with ultra-pure water as appropriate to ensure optimal scattering intensity and to minimize multiple scattering effects. The mean particle diameter (Z-average) and polydispersity index (PDI) were recorded for each sample to evaluate particle size distribution and formulation uniformity. Measurements were conducted using backscattering at an angle of 173°, measurement temperature was set at 25 °C, and each sample was analyzed in triplicate to ensure reproducibility. The results were reported as mean ± standard deviation. Acceptance criteria for the formulation were defined as a mean particle diameter of <20 nm and a PDI < 0.2, consistent with the target profile for stable and uniform Deciparticle™ formation.

Formulation optimization was conducted to evaluate the effect of API-to-polymer ratio on particle formation and stability. Everolimus purchased from BrightGene Pharma (Chongqing, China) and mPEG-Chol were each prepared separately in ethanol at a concentration of 10 mg/mL and vortexed to ensure complete dissolution. A series of formulations were prepared in 1.5 mL tubes by mixing fixed volumes of Everolimus solution (0.1 mL) with increasing volumes of mPEG-Chol polymer solution to achieve API-to-polymer ratios ranging from 1:1 to 1:10. Each mixture was vortexed for 15–20 s to ensure homogeneity. Ethanol was subsequently removed by evaporation on a 60 °C heat block. Following solvent removal, the samples were allowed to cool to room temperature (RT) for approximately 15 min. The resulting films were reconstituted with 250 µL of Water for Injection (WFI) to achieve a final Everolimus concentration of 4 mg/mL and gently mixed at RT for 15 min to facilitate micelle formation.

Scale-up productions were performed by dissolving mPEG-Chol in absolute ethanol, and the polymer/ethanol solution was used to dissolve the desired amount of Everolimus. After the organic solution was fully mixed, it was resolubilized with a pre-made lactose solution (Spectrum Chemical, Gardena, CA, USA) in Water for Injection (Cytiva, Marlborough, MA, USA) to achieve a final Everolimus concentration of 4 mg/mL at room temperature. Once fully resolubilized, the solution was filtered through a 0.22 µm PES filter to ensure sterility and uniformity. The filtered solution was then filled into vials and subjected to a 5-day lyophilization cycle. The final drug product was stored at 2–8 °C and protected from light. All procedures should be conducted under amber lighting conditions, as Everolimus is light-sensitive.

Formulations were evaluated for physical appearance (clarity and precipitation) and particle size characteristics using dynamic light scattering (DLS). The mean particle diameter (Z-average) and polydispersity index (PDI) were measured at 0 h and 24 h after storage at RT to assess initial formation and short-term stability.

### 4.4. Plasma Protein Binding of Sapu003

Plasma protein binding of Sapu003 (Everolimus for Injection) and reference Everolimus was evaluated using a validated ultrafiltration method in plasma obtained from CD-1 mouse, SD rat, beagle dog, cynomolgus monkey, and human donors. The study was performed using LC-MS/MS quantitation. Sapu003 consisted of a lyophilized Everolimus formulation (MW 958.22 g/mol) reconstituted in saline. Test concentrations were 0.200, 2.00, and 20.0 μM. These concentrations correspond to 0.000192, 0.00192, 0.0192 mg/mL.

Plasma samples were thawed at 37 °C and centrifuged to remove particulates prior to use. The test articles were added to plasma and incubated at 37 °C for 5 h. Following the equilibration, the samples were processed using Amicon^®^ Ultra-0.5 centrifugal ultrafiltration devices. Drug concentrations in the ultrafiltrate (free fraction) and plasma compartment (total fraction) were quantified through LC-MS/MS. Warfarin (1 μM) served as the positive control to confirm assay performance. The positive control warfarin demonstrated expected species-dependent plasma protein binding, confirming proper assay performance and reliability of the ultrafiltration methodology. Recovery and stability values remained within acceptable ranges throughout the study.

The assay was designed to determine the fraction of freely diffusible Everolimus present following dilution into plasma. During ultrafiltration, free drug passes through the ultrafiltration membrane whereas protein-associated or nanoparticle-associated drug is retained. Accordingly, the assay provides an assessment of plasma association and dilution stability under physiologically relevant conditions.

The lower limit of quantitation (LLOQ) for Everolimus was 1.00 nM. Values below this threshold were reported as below quantifiable limits (<1.00 nM). For Everolimus (MW 958.22 g/mol), the LLOQ corresponds to: 1.00 nM/0.000958 μg/mL/0.000000958 mg/mL. Fraction unbound (fu) and plasma protein binding (PPB) were calculated.

### 4.5. Sapu003 Clinical Production

Everolimus (API) (BrightGene Pharma, China) was used as the active pharmaceutical ingredient for preparation of the Sapu003 nanoparticle formulation. The amphiphilic carrier polymer mPEG-Chol was used as the nanoparticle-forming excipient. Absolute ethanol (200 proof) (Thermo Fisher Scientific, Waltham, MA, USA) served as the organic solvent for drug–polymer dissolution. Anhydrous Lactose (Spectrum Chemical, New Brunswick, NJ, USA) was used as a stabilizing excipient. Water for Injection (WFI) (Cytiva, Marlborough, MA, USA) was used for preparation of the aqueous phase. All reagents were of pharmaceutical or analytical grade and used as received. All processing was performed under controlled lighting conditions to minimize photodegradation of Everolimus.

Sapu003/Everolimus for Injection bulk nanoparticle formulation was manufactured under GMP environment. The target formulation strength of the bulk dispersion was 4.0 mg/mL Everolimus, corresponding to a 12 g drug load per batch. The manufacturing process consisted of polymer melting, preparation of a drug–polymer solution in ethanol, formation of nanoparticles through controlled aqueous dispersion, bulk homogenization, sterile filtration, and refrigerated storage of the final bulk formulation.

Everolimus was added to the polymer–ethanol solution under amber light conditions to minimize potential photodegradation. The mixture was vortex mixed and subsequently agitated on an orbital shaker until the drug was completely dissolved and a homogeneous solution was obtained. The resulting drug–polymer solution was transferred to a sterile reaction flask for nanoparticle formation.

An aqueous lactose-stabilizing solution was prepared in a separate sterile vessel. Water for Injection was combined with anhydrous lactose. The solution was stirred on a magnetic stir plate for approximately 10 min to reach homogeneity. Nanoparticle formation was initiated by slowly adding the aqueous lactose solution to the drug–polymer ethanol solution under continuous mixing. The mixture was rotated and mixed during which nanoparticle formation occurred and the dispersion became visually clear. This process produced a homogeneous bulk nanoparticle dispersion containing Everolimus.

The bulk nanoparticle dispersion was subjected to sterile filtration using non-pyrogenic sterile vacuum filtration units. Process recovery of approximately 96–100% relative to the theoretical batch mass. Following sterile filtration, the bulk formulation was aseptically filled into depyrogenated vials and lyophilized to produce the final drug product. Lactose served as a lyo-protectant and bulking agent to preserve nanoparticle integrity during freezing and drying, improve cake structure, and facilitate rapid reconstitution. The final clinical presentation targeted 20 mg Everolimus per vial. Prior to administration, the lyophilized product was reconstituted with Water for Injection to yield a final Everolimus concentration of approximately 4 mg/mL.

Sapu003 drug products were tested though multiple release tests including identity and potency, as well as mean particle diameter and polydispersity index. Everolimus identity and content was quantified using an Agilent 1260 Infinity II HPLC (Santa Clara, CA, USA) with quaternary pump, autosampler, thermostatic column compartment, and VWD. An Agilent Zorbax Eclipse XDB-C18 (Santa Clara, CA, USA), 3 mm × 250 mm reverse phase column was used with aqueous 0.27 g L^−1^ potassium phosphate and 100% methanol chosen as the weak and strong mobile phases respectively. The samples were diluted to a theoretical Everolimus concentration of 20 µg mL^−1^ in 40% aqueous acetonitrile and stored in amber 2 mL HPLC vials at 4 °C for no more than 48 h. The absorbance of Everolimus and its Oxepane isomer were measured at 275 nm at a flow rate of 1.1 mL min^−1^ and a gradient method. Potency was determined by single-point calibration using a working standard provided by the US Pharmacopeia (North Bethesda, MD, USA). The Z-average particle diameter and polydispersity index of the micelle nanoparticles were determined through dynamic light scattering (Malvern Zetasizer, ZEN3600) at an angle of 173° at room temperature. Drug products were reconstituted in ultra-pure water and diluted 10-fold before analysis.

The stability of Sapu003 formulations was evaluated under both storage and in-use conditions using particle size (Z-average) and polydispersity index (PDI) as critical quality attributes. Chemical stability was also evaluated via HPLC, pH measurement and water content testing. For storage stability, lyophilized or formulated samples were stored under the following conditions: 2–8 °C, 25 °C/60% RH ± 5% RH, and 30 °C/65% RH ± 5% RH. At each condition, samples were taken out from the stability chambers and undergo predefined tests. Everolimus assay percent was tested using Agilent 1260 Infinity II HPLC; water content percent was tested via Karl Fischer Titrator (Mettler Toledo, Columbus, OH, USA). For solution acidity and particle size distribution, the samples were reconstituted at predefined time points and analyzed using pH meter and dynamic light scattering (DLS) respectively. For in-use stability, formulations were first reconstituted in Water for Injection (WFI) and evaluated immediately (0 h), followed by storage under the same three temperature conditions (2–8 °C, 25 °C/60% RH ± 5% RH, and 30 °C/65% RH ± 5% RH). Particle size and PDI were measured at 0 h, 24 h, 7 days, and 14 days post-reconstitution. Stability was assessed based on the ability of the formulation to maintain particle size within the predefined acceptance criteria (<20 nm) and PDI < 0.2, indicating preservation of micelle integrity and formulation homogeneity over time under the tested conditions.

### 4.6. Residual Solvent Analysis of Sapu003 Drug Product

Residual solvent content in Sapu003/Everolimus for Injection was evaluated using a validated static headspace gas chromatography method with flame ionization detection (HS-GC/FID) in accordance with USP <467> Residual Solvents and USP <1467> Validation of Residual Solvent Procedures.

Briefly, lyophilized drug product samples were transferred into sealed headspace vials and analyzed using an Agilent 8890 gas chromatograph equipped with a 7697A headspace autosampler (Agilent, Santa Clara, CA, USA) and HP-5 capillary column (30 m × 0.32 mm, 0.25 μm film thickness). Headspace equilibration and chromatographic conditions were optimized for the detection and quantitation of residual organic solvents potentially introduced during manufacture, including ethanol, methanol, acetonitrile, dichloromethane, toluene, acetone, ethyl acetate, and n-heptane.

Quantitation was performed using external calibration standards prepared gravimetrically across the validated concentration range. Calibration curves demonstrated linearity with correlation coefficients ≥ 0.99. Method validation included assessments of specificity, linearity, accuracy, precision, robustness, carry-over, and sample stability. Spike recovery studies demonstrated recoveries within 80–120%, while repeatability and intermediate precision studies showed relative standard deviations generally below 3%. No interfering peaks were observed at the retention times of the target analytes, confirming method specificity.

System suitability was verified before each analytical sequence using replicate injections of reference standards. Acceptance criteria included peak-area relative standard deviation ≤ 2.0%, acceptable chromatographic resolution, and signal-to-noise ratios ≥ 10 at the limit of quantitation. Carry-over was assessed using high-concentration solvent standards followed by blank injections and demonstrated no detectable residual signal. Sample and standard preparations were confirmed to be stable throughout the analytical period.

Residual solvent concentrations were calculated from the corresponding calibration curves and reported in parts per million (ppm). Acceptance criteria were established according to USP <467> and ICH Q3C guidelines. Class 2 solvents were controlled according to their respective permitted daily exposure limits, including methanol (3000 ppm), acetonitrile (410 ppm), dichloromethane (600 ppm), and toluene (890 ppm). Class 3 solvents, including ethanol, acetone, ethyl acetate, and n-heptane, were controlled to not more than 5000 ppm unless otherwise justified. All tested lots were required to comply with the applicable residual solvent limits prior to release.

### 4.7. Sapu003 Formulation Space and In-Use Stability Evaluation

Statistical evaluation of both formulation design space and in-use stability for the Sapu003 drug product was performed using Design-Expert (version 25.0.6.0, Stat-Ease Inc., Minneapolis, MN, USA). The objective was to systematically assess the effects of formulation variables and in-use conditions on mean particle size (nm). For formulation space optimization, a design of experiments (DoE) approach was applied in which key independent variables, including operating concentration and API-to-polymer ratio, were evaluated across predefined ranges. For in-use stability analysis, the impact of storage and handling conditions—such as in-use time and operating temperature were examined. The data were similarly analyzed using regression modeling and ANOVA to identify significant factors affecting stability. Response surface plots were used to characterize condition-dependent stability behavior and to define acceptable ranges for in-use conditions.

### 4.8. Xenograft Studies

All in vivo studies were conducted at a GLP-certified contract development and manufacturing organization (Shanghai, China) in accordance with institutional standard operating procedures and relevant regulatory guidance. The study protocols were reviewed and approved by the Institutional Animal Care and Use Committee (IACUC) before animal receipt or transfer.

Tumor cells were maintained in MEM medium with 10% FBS. Cells were incubated at 37 °C in a humidified incubator with 5% CO_2_. Xenograft model was established by inoculating tumor cells (3 × 10^6^ cells/0.1 mL/animal, with 50% Matrigel (Corning Inc., New York, NY, USA) into the right flank of the BALB/c nude mice under sterile conditions. Tumor-inoculated mice were randomized into different groups and received different treatments. The tumor sizes and animal body weights were measured twice per week. The tumors were measured using a caliper. Tumor volumes were estimated from measurements of the two diameters of the individual tumors. Clinical signs were recorded daily. Animal survivals were recorded. Observations included general health, body weight, behavior, and any adverse effects related to the dose administration.

Tumor volume (TV, mm^3^) was calculated as TV = (axb^2^)/2, as the tumor length and b as the tumor width; percent body weight change (%BWC) was calculated as %BWC = (BW_t_ − BW_0_)/BW_0_ 100%, with BW_t_ as the body weight on measurement day and BW_0_ as the body weight on randomization day. All quantitative data in the study were expressed as mean ± SEM (standard error of means). The tumor growth curves of test articles were plotted with the observation time on the *X*-axis, and corresponding tumor volume (geometric mean) on the *Y*-axis. The body weight change in test articles was plotted with the observation time on the *X*-axis and corresponding body weight (geometric mean) on the *Y*-axis.

## 5. Conclusions

Through screening a 38-member mPEG-block polymer library, mPEG-Chol was identified as a lead candidate capable of forming stable Deciparticles™ under 20 nm with Everolimus and demonstrating robust antitumor activity across multiple xenograft models. mPEG-Chol, representing the broad chemical space of the Deciparticle™ platform, enables a reproducible cGMP manufacturing process and long-term drug stability. These findings support the ongoing first-in-human study of intravenously administered Everolimus via weekly Sapu003/Everolimus for Injection and highlight the Deciparticle™ technology as a versatile platform for delivering other challenging, water-insoluble oncology agents.

## Figures and Tables

**Table 1:** ANOVA table for the DoE experiment.

Source	Sum of Squares	df	Mean Square	F-Value	*p*-Value
Model	3.399 × 10^5^	10	33,986.19	21.46	<0.0001
A-concentration	6077.31	1	6077.31	3.84	0.0575
B-ratio	10,166.89	1	10,166.89	6.42	0.0155
C-ethanol	4097.69	1	4097.69	2.59	0.1160
D-lactose	2975.49	1	2975.49	1.88	0.1785
AB	1583.80	1	1583.80	1.00	0.3236
AC	2734.39	1	2734.39	1.73	0.1967
AD	5856.07	1	5856.07	3.70	0.0620
BC	2070.98	1	2070.98	1.31	0.2599
BD	2324.00	1	2324.00	1.47	0.2332
CD	3172.92	1	3172.92	2.00	0.1651
Residual	60,173.14	38	1583.50		
Lack of fit	60,066.58	26	2310.25	260.15	<0.0001
Pure error	106.56	12	8.88		
Cor total	4.000 × 10^5^	48			

**Table 2:** LogP value for the compounds.

Class	Molecule	Reported Value	Descriptor/Source
Rapalog	Sirolimus	6.0	XLogP3-AA/PubChem
Rapalog	Everolimus	5.9	XLogP3-AA/PubChem
Rapalog	Temsirolimus	5.6	XLogP3-AA/PubChem
Rapalog	Ridaforolimus	5.9	XLogP3/Pharma Compass
Rapalog	Umirolimus (Biolimus A9)	6.8	XLogP3-AA/PubChem
Ascomycin	Tacrolimus	2.7	XLogP3/PubChem
Ascomycin	Pimecrolimus	3.8	XLogP3/Pharma Compass
Cyclic peptide	Cyclosporine A	7.5	XLogP3-AA/PubChem
Peptide	Exenatide	−21.0	XLogP3-AA/PubChem
Peptide–lipid conjugate	Liraglutide	−3.4	XLogP3-AA/PubChem
Peptide–lipid/PEG conjugate	Semaglutide	−5.8	XLogP3-AA/PubChem
Fc-fusion peptide	Dulaglutide	−14.3	XLogP3-AA/PubChem
Peptide	Lixisenatide	−30.8	XLogP3-AA/PubChem

**Table 3:** Summary results of Sapu003 release tests and storage stability evaluation.

Batch ID	Storage Condition	Storage Time (Month)	Assay (%), Spec (95–102%)	Residual Ethanol (ppm), Spec (≤5000 ppm)	Average Particle Size (nm), Spec (<30 nm)	pH, Spec (4.0–7.0)	Water Content (%), Spec (≤3%)
CTM25015	Release	Initial	96.1	115.7	13.5	5.2	1.5
	2–8 °C	1 M	96.1	—	13.7	5.5	1.3
		3 M	96.6		23.5	5.5	2.0
		6 M	93.5		15.5	5.1	0.8
CTM25015	25 °C/60% RH ± 5% RH	1 M	94.8		14.1	5.1	1.9
		3 M	91.2		22.2	5.4	1.7
		6 M	87.9		13.8	5.2	1.3
CTM25015	30 °C/65% RH ± 5% RH	1 M	90.6		20.7	4.9	1.7
		3 M	92.1		15.6	4.9	1.5
		6 M	59.5		14.5	4.1	0.7
CTM25016	Release	Initial	98.4	101.0	13.9	5.2	1.4
	2–8 °C	1 M	95.6	—	12.1	5.0	1.6
		3 M	97.6		18.7	5.5	1.5
		6 M	98.7		15.6	4.9	1.3
CTM25016	25 °C/60% RH ± 5% RH	1 M	94.2		22.5	5.0	1.4
		3 M	97.3		13.0	5.3	1.8
		6 M	95.2		15.3	4.8	1.3
CTM25016	30 °C/65% RH ± 5% RH	1 M	90.3		26.8	5.4	1.3
		3 M	88.7		14.6	4.8	1.6
		6 M	68.6		12.7	4.1	0.9
CTM25017	Release	Initial	94.5	75.6	13.1	5.0	1.6
	2–8 °C	1 M	98.8	—	18.5	5.2	1.8
		3 M	97.4		16.3	5.4	2.5
		6 M	97.0		15.0	5.0	0.9
CTM25017	25 °C/60% RH ± 5% RH	1 M	98.4		17.2	5.0	2.0
		3 M	94.1		14.9	5.2	1.8
		6 M	92.0		14.5	4.7	1.2
CTM25017	30 °C/65% RH ± 5% RH	1 M	93.8		16.6	5.1	1.5
		3 M	88.0		14.8	4.9	1.8
		6 M	79.5		13.0	4.5	1.0

**Table 4:** ANOVA table for Sapu003 in-use stability analysis.

Source	Sum of Squares	df	Mean Square	F-Value	*p*-Value
Model	2453.52	29	84.60	4.69	<0.0001
A-A	371.86	3	123.95	6.87	0.0002
B-storage time	19.74	1	19.74	1.09	0.2963
C-storage temperature	88.73	1	88.73	4.91	0.0272
D-in-use time	740.86	1	740.86	41.04	<0.0001
E-in-use temperature	243.28	1	243.28	13.48	0.0003
AB	53.63	3	17.88	0.9903	0.3973
AC	54.59	3	18.20	1.01	0.3892
AD	25.09	3	8.36	0.4633	0.7081
AE	19.58	3	6.53	0.3616	0.7808
BC	100.46	1	100.46	5.57	0.0188
BD	65.52	1	65.52	3.63	0.0575
BE	160.26	1	160.26	8.88	0.0031
CD	88.49	1	88.49	4.90	0.0274
CE	20.90	1	20.90	1.16	0.2826
DE	131.04	1	131.04	7.26	0.0074
B^2^	224.68	1	224.68	12.45	0.0005
C^2^	4.29	1	4.29	0.2377	0.6261
D^2^	51.95	1	51.95	2.88	0.0906
E^2^	1.53	1	1.53	0.0847	0.7702
Residual	6968.21	386	18.05		
Lack of fit	6968.21	380	18.34		
Pure error	0.0000	6	0.0000		
Cor total	9421.73	415			

## Data Availability

The datasets generated and analyzed during the current study are not publicly available due to proprietary and confidential business information but are available from the corresponding author upon reasonable request and subject to approval by the study sponsor.

## Abbreviation

The following abbreviation is used in this manuscript:

API	active pharmaceutical ingredient
DoE	design of experiments
ISO	International Organization for Standardization
GMP	Good Manufacturing Practices

## References

[1] Gabardi S., Baroletti S.A. (2010). Everolimus: A proliferation signal inhibitor with clinical applications in organ transplantation, oncology, and cardiology. Pharmacotherapy.

[2] Rouf M.A., Vural I., Renoir J.M., Hincal A.A. (2009). Development and characterization of liposomal formulations for rapamycin delivery and investigation of their antiproliferative effect on MCF7 cells. J. Liposome Res..

[3] Liggins R.T., Burt H.M. (2002). Polyether-polyester diblock copolymers for the preparation of paclitaxel-loaded polymeric micelle formulations. Adv. Drug Deliv. Rev..

[4] Rao D.A., Nguyen D.X., Mishra G.P., Doddapaneni B.S., Alani A.W.G. (2015). Preparation and characterization of individual and multi-drug loaded physically entrapped polymeric micelles. J. Vis. Exp..

[5] Mishra G.P., Doddapaneni B.S., Nguyen D.X., Alani A.W.G. (2014). Antiangiogenic effect of docetaxel and everolimus as individual and dual-drug-loaded micellar nanocarriers. Pharm. Res..

[6] Gianessi L., Magini A., Dominici R., Giovagnoli S., Dolcetta D. (2023). A stable micellar formulation of RAD001 for intracerebroventricular delivery and the treatment of Alzheimer’s disease and other neurological disorders. Int. J. Mol. Sci..

[7] (2015). ISO 5 Cleanroom Standard.

[8] (1978). Current Good Manufacturing Practice in Manufacturing Processing, Packing, or Holding of Drugs.

[9] Zhu S., Lansakara-P D.S.P., Li X., Cui Z. (2012). Lysosomal delivery of a lipophilic gemcitabine prodrug using novel acid-sensitive micelles improved its antitumor activity. Bioconjug. Chem..

[10] Diezi T.A., Takemoto J.K., Davies N.M., Kwon G.S. (2011). Pharmacokinetics and nephrotoxicity of amphotericin B-incorporated poly (ethylene glycol)-block-poly (N-hexyl stearate L-aspartamide) micelles. J. Pharm. Sci..

[11] Lavasanifar A., Samuel J., Kwon G.S. (2001). Poly (ethylene oxide)-block-poly (N-hexyl stearate L-aspartamide) micelles for drug delivery: Formulation and in vitro characterization of amphotericin B. J. Control. Release.

[12] Dong K., Liu Z., Li Z., Ren J., Qu X. (2016). Biodegradable mixed MPEG-SS-2SA/TPGS micelles for triggered intracellular release of paclitaxel and reversing multidrug resistance. Int. J. Nanomed..

[13] Guan Q., Sun S., Li X., Lv S., Wang Y., Wang L., Song X. (2017). Docetaxel-loaded self-assembly stearic acid-modified Bletilla striata polysaccharide micelles and their anticancer effect: Preparation, characterization, cellular uptake and in vitro evaluation. Cellulose.

[14] Yu Y., He Y., Xu B., Zhang Q., Wang X., Zhang Z. (2013). Self-assembled methoxy poly (ethylene glycol)-cholesterol micelles for hydrophobic drug delivery. J. Pharm. Sci..

[15] He Z.Y., Chu B.Y., Wei X.W., Li J., Edwards C.K., Chen Y.C. (2014). Recent development of poly (ethylene glycol)-cholesterol conjugates as drug delivery systems. Int. J. Pharm..

[16] Búzová D., Kasák P., Miškovský P., Jancura D. (2013). Solubilization of poorly soluble photosensitizer hypericin by polymeric micelles and polyethylene glycol. Gen. Physiol. Biophys..

[17] Ma Q., Li B., Yu Y., Zhang Y., Wu Y., Ren W., Zheng Y., He J., Xie Y., Song X. (2013). Development of a novel biocompatible poly (ethylene glycol)-block-poly(γ-cholesterol-l-glutamate) as hydrophobic drug carrier. Int. J. Pharm..

[18] Chang W., Olivar E., Pai G., Low J., Min S., Hoff R., Chang N., Hoque T., Park A., Lee C. (2026). Abstract PS4-06-05: Sapu003: Everolimus for Injection—Pharmacokinetic Rationale for Phase I Evaluation in HR^+^/HER2^−^ Metastatic Breast Cancer. Clin. Cancer Res..

[19] Yang D.B., Zhu J.B., Huang Z.J., Wang Y., Zhu J.L. (2008). Synthesis and application of poly (ethylene glycol)-cholesterol conjugates in physicochemical characterization of nonionic surfactant vesicles. Colloids Surf. B Biointerfaces.

[20] Sant V.P., Nagarsenker M.S. (2011). Synthesis of monomethoxypolyethyleneglycol-cholesteryl ester and effect of its incorporation in liposomes. AAPS PharmSciTech.

